# Expression of C4.4A in an In Vitro Human Tissue-Engineered Skin Model

**DOI:** 10.1155/2017/2403072

**Published:** 2017-09-07

**Authors:** Benedikte Jacobsen, Danielle Larouche, Olivier Rochette-Drouin, Michael Ploug, Lucie Germain

**Affiliations:** ^1^The Finsen Laboratory, Copenhagen University Hospital (Rigshospitalet), Copenhagen Biocenter, Copenhagen, Denmark; ^2^Biotech Research and Innovation Centre (BRIC), University of Copenhagen, Copenhagen, Denmark; ^3^Centre de Recherche du CHU de Québec-Université Laval, Department of Surgery, Faculty of Medicine, Université Laval and Centre de Recherche en Organogénèse Expérimentale de l'Université Laval (LOEX), Québec, QC, Canada

## Abstract

A multi-LU-domain-containing protein denoted C4.4A exhibits a tightly regulated membrane-associated expression in the suprabasal layers of stratified squamous epithelia such as skin and the esophagus, and the expression of C4.4A is dysregulated in various pathological conditions. However, the biological function of C4.4A remains unknown. To enable further studies, we evaluated the expression of C4.4A in monolayer cultures of normal human keratinocytes and in tissue-engineered skin substitutes (TESs) produced by the self-assembly approach, which allow the formation of a fully differentiated epidermis tissue. Results showed that, in monolayer, C4.4A was highly expressed in the centre of keratinocyte colonies at cell-cell contacts areas, while some cells located at the periphery presented little C4.4A expression. In TES, emergence of C4.4A expression coincided with the formation of the* stratum spinosum.* After the creation of a wound within the TES, C4.4A expression was observed in the suprabasal keratinocytes of the migrating epithelium, with the exception of the foremost leading keratinocytes, which were negative for C4.4A. Our results are consistent with previous data in mouse embryogenesis and wound healing. Based on these findings, we conclude that this human TES model provides an excellent surrogate for studies of C4.4A and Haldisin expressions in human stratified epithelia.

## 1. Introduction

The stratified squamous epithelium of human epidermis represents a highly complex organ, which provides an important protective barrier against opportunistic pathogens and dehydration. Several proteins belonging to the Ly6/uPAR/*α*-neurotoxin (LU) protein domain family [1] are expressed by epidermal keratinocytes, including E48, SLURP 1, C4.4A, and Haldisin [2]. LU domain-containing proteins have evolved to serve multiple diverse physiologic functions, for example, inhibition of autologous complement activation, CD59 [3]; driving cell surface-associated plasminogen activation, uPAR [4]; localizing intravascular triglyceride hydrolysis on capillaries, GPIHBP1 [5, 6]; regulating fertility, TEX101 [7]; and neutrophil function, CD177 [8]. It is noteworthy that maintenance of epidermal homeostasis is compromised by missense mutations or congenital deficiency of one of the LU proteins (SLURP1: secreted Ly6/uPAR protein 1), which leads to hyperkeratosis and palmoplantar keratoderma—a disorder known as Mal de Meleda [9, 10].

We have previously reported that two genes,* LYPD3 *and* LYPD5—*located in the same small gene cluster as* CD177*,* TEX101,* and* PLAUR* on chromosome 19q13 [11] and encoding the LU proteins C4.4A and Haldisin—constitute a new set of surface-exposed histological biomarkers for squamous epithelia differentiation: C4.4A being confined to* stratum spinosum *[12] and Haldisin to* stratum granulosum* [13]. Both proteins are glycosyl-phosphatidyl-inositol- (GPI-) anchored membrane glycoproteins with two LU domains [13–15]. The strict regulation of C4.4A expression is clearly illustrated by immunohistochemical stainings of epithelial transition zones such as the uterovaginal and anorectal junctions, where C4.4A displays an all-or-nothing shift in its expression pattern at the border between squamous and columnar epithelia [12].

An early study comparing rat pancreatic adenocarcinoma cell lines proposed a link between C4.4A and metastasis [16]. Prompted by this intriguing association, several subsequent studies independently reported that high C4.4A expression in solid cancer lesions of patients suffering from pulmonary adenocarcinoma [17–20], esophageal squamous cell carcinoma [21, 22], and colorectal adenocarcinoma [23] generally correlates to poor patient survival. During a comprehensive survey of a number of invasive and noninvasive skin lesions [24], we observed that C4.4A was only upregulated in the front of invasive lesions, and this occurred independently of whether they were malignant (squamous cell carcinomas) or benign (keratoacanthomas). In the normal skin, C4.4A expression is predominantly linked to keratins (K) 1 and 10, but, in the invasive lesions, it shifts to K5 and K14—which is indicative of a lower differentiation state of the invading keratinocytes [24]. Along the same lines, the deep invasive regions of human esophageal squamous cell carcinomas present a pronounced C4.4A expression [21]. Migrating keratinocytes also express significant amounts of C4.4A during reepithelization of incisional murine skin wounds [15].

Despite this clear impact as robust biomarkers for stratified squamous cell differentiation and prognostic impact on particularly pulmonary adenocarcinoma patient survival, the functional role of C4.4A in maintaining epithelial integrity and promoting invasion remains enigmatic. Mice deficient in C4.4A expression by* Lypd3* gene ablation have only modest overt phenotypes [25]. To be able to supplement studies on this genetic mouse model with a human counterpart, we therefore characterize the expression of C4.4A in a surrogate in vitro model of human skin produced by the self-assembly approach of tissue engineering [26, 27]. The development of the epidermis into a fully stratified four-layer squamous epithelium, including the basal, spinous, granular, and corneal layers, can be closely monitored as a function of time in this human tissue-engineered skin substitute (TES) [28, 29].

## 2. Materials and Methods

The institution's committee for the protection of human subjects approved the study. All procedures followed were in accordance with the Helsinki Declaration of 1975.

### 2.1. Cell Culture

Fibroblasts were isolated from human skin biopsies obtained from reductive breast surgery (21-year-old) and foreskin (3-day-old). Keratinocytes were isolated from foreskin, breast, and facelift surgeries (4-day-old, 61- and 55-year-old, resp.). All biopsies were obtained after informed consent was given. Fibroblasts and keratinocytes were extracted, cultured, cryopreserved, and thawed as described [30]. Fibroblasts were cultured in Dulbecco's modified Eagle's medium (DMEM; Invitrogen) with 10% fetal calf serum (HyClone). Keratinocytes were grown on a feeder layer of irradiated murine 3T3 fibroblasts as described [31] and cultured in DMEM/Ham's F12 medium (Invitrogen) in a 3 : 1 proportion, supplemented with 24.3 *μ*g/ml adenine (Sigma-Aldrich, ON, Canada), 5% FetalClone II (HyClone), 5 *μ*g/ml insulin (Sigma-Aldrich), 0.4 *μ*g/ml hydrocortisone (Calbiochem), 0.1 nM cholera toxin (Sigma-Aldrich), and 10 ng/ml epidermal growth factor (Austral Biologicals). Antibiotics (100 IU/ml penicillin G and 25 *μ*g/ml gentamicin [BD Bioscience]) were added to both culture media. For immunofluorescence, keratinocytes and 3T3 were grown on coverslips (22 × 22 mm; Fisher Scientific) in 6-well plates until 80% confluence. All cultures were kept at 37°C in a humidified incubator containing 8% CO_2_, and the culture medium was changed three times per week.

### 2.2. Production of Human Tissue-Engineered Skin

The method of reconstruction of human skin by the self-assembly approach of tissue engineering has been described in detail elsewhere [32]. Briefly, human fibroblasts were cultured 28 days in medium containing 50 *μ*g/ml ascorbic acid, which promotes the secretion of extracellular matrix, leading to the organization of cells into manipulable tissue sheets. Two of these sheets were stacked to make up the dermal portion of the TES. After one week of culture, allowing the fusion of the sheets, keratinocytes were seeded on top of these and kept in submersion for another week. The process of epidermal differentiation of the keratinocytes was initiated by raising the TES to the air-liquid (A/L) interface. Biopsies were taken every day for the first 14 days of culture at the A/L interface. Tissue samples were embedded in optimal cutting temperature (OCT) compound, frozen in liquid nitrogen, and stored at −80°C until analysis.

### 2.3. Wound Healing in Human Tissue-Engineered Skin

At day 11 A/L, mature TES composed of newborn keratinocytes (4-day-old) and adult fibroblasts (21-day-old) was wounded by a 6 mm punch biopsy (Acuderm Inc.) as described previously [33]. Migration of the keratinocytes for reepithelialization of the wound was allowed by placing the wounded skin on a third fibroblast sheet produced as described above. The healing process was followed microscopically by biopsy collection at different time points.

### 2.4. Immunofluorescence Stainings

Adult and newborn keratinocytes in monolayer culture were grown on coverslips (22 × 22 mm; Fisher Scientific) in 6-well plates until they proliferated to 80% confluence. Cells were rinsed in phosphate buffered saline (PBS), permeabilized in 99% methanol at −20°C for 10 min, and conserved in PBS. Frozen tissue embedded in OCT was sectioned at 5 *μ*m, fixed in acetone at −20°C for 10 min, and rinsed in PBS. For immunofluorescence staining, slides or coverslips were incubated with primary antibodies (25 *μ*l/section) for 45 min at room temperature and with Alexa Fluor dye-conjugated secondary antibodies for 30 min in darkness (25 *μ*l/section). For double immunofluorescence, the two primary antibodies were added simultaneously to the sections and detected with species-specific anti-IgG antibodies. Cell nuclei were visualized with Hoechst 33258 (Sigma) and the sections mounted in PBS/glycerol/gelatin (pH 7.6). The primary antibodies used in this study were rabbit polyclonal antibodies (pAbs) raised against a recombinant secreted version of human C4.4A, Haldisin [13, 15, 34] and K14 peptide [35], and mouse monoclonal antibodies recognizing domains I + II of C4.4A [21], K10 clone RKSE60 (Cedarlane Laboratories), laminin-5 *γ*2 (Millipore), *β*-catenin (Santa Cruz), and anti-*α*3 subunit of integrin clone HB-8530 (VM2). Goat anti-rabbit IgG coupled to Alexa 488 and anti-mouse IgG coupled to Alexa 594 (Molecular Probes) were used as secondary antibodies, yielding green and red fluorescence signals, respectively. Negative controls consisted in omitting the primary antibodies in the staining procedure. Fluorescence and phase contrast images were recorded using a Zeiss Axio Imager M2 microscope (Carl Zeiss Canada Ltd.).

## 3. Results

### 3.1. C4.4A Expression in Keratinocytes Grown in Monolayer

**Figure 1:**
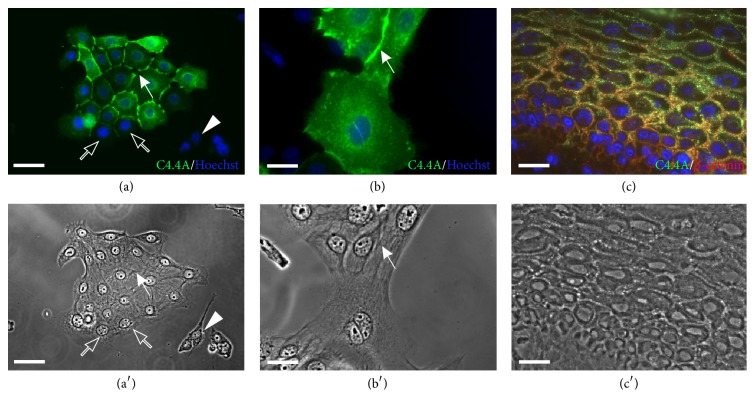
C4.4A in monolayer culture of human keratinocytes. (a, b) C4.4A expression in cultured human keratinocytes. Confinement of C4.4A to points of cell-cell contact is emphasized by arrows. (c) Colocalization of C4.4A (green) with the adherens junction protein *β*-catenin (red) in TES at day 28 of culture at the A/L interface. Corresponding phase contrast pictures are depicted in panels (a′), (b′), and (c′), respectively. Cell nuclei are stained with Hoechst (blue). Note that murine 3T3 fibroblasts were devoid of C4.4A expression (arrowhead). Open arrows point toward less differentiated keratinocytes at the periphery of the colony, which are not labeled for C4.4A. A/L, air-liquid; d, day; TES, tissue-engineered skin. Scale bar: (a) and (a′), 50 *μ*m; (b), (b′), (c), and (c′), 25 *μ*m.

To check the C4.4A expression pattern in human keratinocytes before their seeding on fibroblast sheets for subsequent squamous differentiation within TES, keratinocytes grown in monolayer were immunolabeled with our anti-C4.4A pAb [15]. The results showed that some keratinocytes in monolayer cultures highly expressed C4.4A at cell-cell borders (Figures 1(a)-1(b), arrows), while the murine 3T3 fibroblasts, used as a feeder layer in the culture, were devoid of reactivity (Figure 1(a), arrowhead). It is interesting to note that some of the less differentiated keratinocytes at the periphery of isolated colonies were not labeled (Figure 1(a), open arrows). Although C4.4A in general was found at the cell membrane, it was nonetheless predominantly confined to those regions forming cell-cell contact points. As evident from double immunofluorescence staining of TES at day 28 A/L, C4.4A colocalized with the adherens junction protein *β*-catenin (Figure 1(c)). Donor age did not modify the C4.4A expression pattern in keratinocytes.

### 3.2. C4.4A Expression in Human Tissue-Engineered Skin

**Figure 2:**
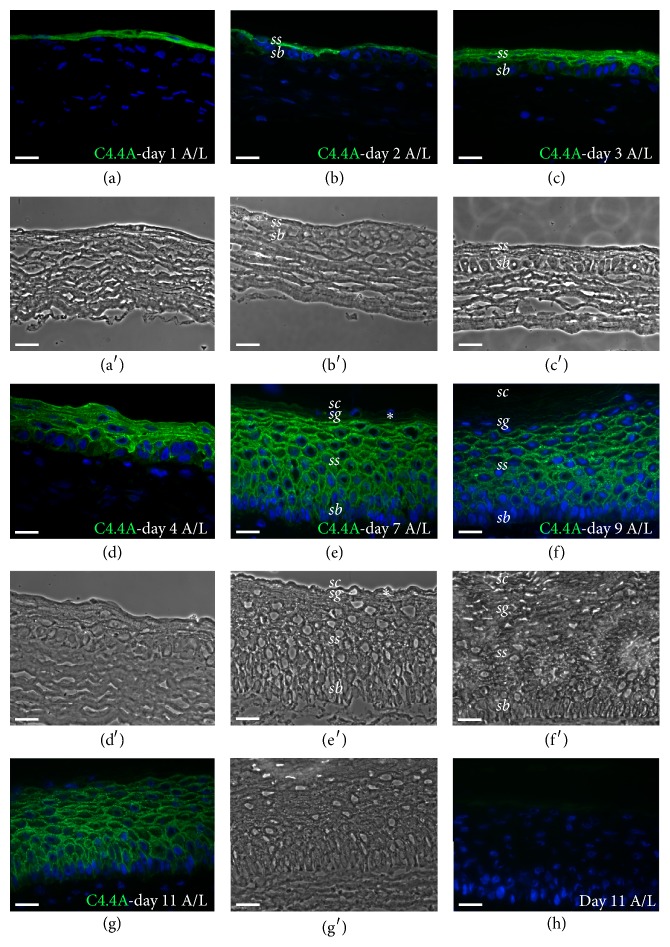
C4.4A in the development of human tissue-engineered skin. (a–g) Expression of C4.4.A (green) in TES at days 1 (a), 2 (b), 3 (c), 4 (d), 7 (e), 9 (f), and 11 (g, h) of culture at the A/L interface. (a′), (b′), (c′), (d′), (e′), (f′), and (g′): Phase contrast images corresponding to panels (a), (b), (c), (d), (e), (f), and (g) respectively. (h) Negative control (omission of the primary antibody). Cell nuclei are stained with Hoechst (blue). A/L, air-liquid; d, day;* sb, stratum basale; ss, stratum spinosum; sg, stratum granulosum; sc, stratum corneum;* TES, tissue-engineered skin. Scale bar: 25 *μ*m.

**Figure 3:**
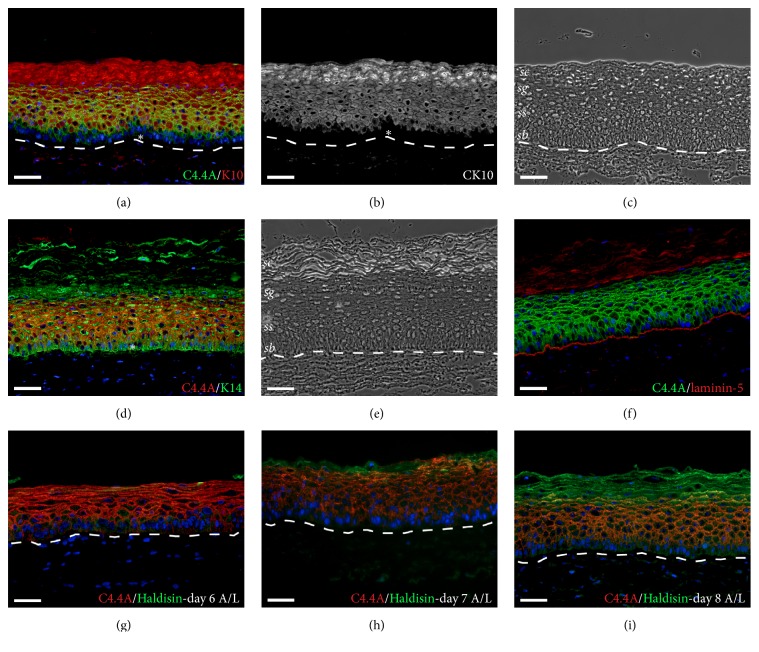
C4.4A expression in relation to epidermal differentiation markers. (a) Expression of C4.4A (green) and K10 (red) in TES at day 10 of culture at the A/L interface. K10 labeling is also shown separately in panel (b). (d) Expression of C4.4A (red) and K14 (green) in TES at day 11 of culture at the A/L interface. (f) Expression of C4.4A (green) and the basement membrane marker laminin-5 (red) in TES at day 11 of culture at the A/L interface. (g–i) Expression of C4.4A (red) and Haldisin (green) in TES at days 6–8 of culture at the A/L interface. (c and e) Phase contrast images corresponding to panels (b) and (d), respectively. Cell nuclei are stained with Hoechst (blue). The difference in K10 and K14 expression in the basal layer is emphasized by asterisks in panels (a), (b), and (d). The localization of the basement membrane, separating the fibroblast sheets of the dermal portion of the TES from the keratinocytes of the epidermal compartment is stressed by a dotted line in panels (a), (b), (c), (e), (g), (h), and (i). A/L, air-liquid; K, keratin;* sb, stratum basale; ss, stratum spinosum; sg, stratum granulosum; sc, stratum corneum;* TES, tissue-engineered skin. Scale bar = 50 *μ*m.

TESs were produced by the self-assembly method of tissue engineering and showed expected characteristics, such as the presence of dermal component covered by an epidermis with the absence of glands and hair follicles (Figures 2 and 3) [27, 32, 36]. To follow the development of a fully stratified squamous epithelium and to determine at which point of the epidermal genesis C4.4A expression is initiated in TESs, biopsies were collected every day after raising the TES to the A/L interface, until day 11, and biopsy sections were immunostained. C4.4A was already present at day 1 A/L (Figure 2(a)) and its expression was maintained throughout the development of the fully stratified epidermis (Figures 2(b)–2(g)). At day 2 of culture at the A/L interface, the staining was confined to suprabasal cells, leaving the* stratum basale *devoid of C4.4A. Of note, the C4.4A expression was almost lost in the higher suprabasal cells around day 7 of culture at the A/L interface (Figure 2(e), asterisk). The* stratum corneum* was also negative for C4.4A, as well as fibroblasts in the underlying dermis. Double immunofluorescence staining for K10, a differentiation marker for keratinocytes present in the suprabasal layers of the epidermis [37], showed that both C4.4A and K10 were absent in the basal layer but expressed in the spinous layer (Figures 3(a)-3(b)). As expected, the anti-K14 reacted with basal keratinocytes that were negative for C4.4A (Figure 3(d)). Given that C4.4A is absent in* stratum basale*, there was no contact with the laminin-5-expressing basement membrane (Figure 3(f)). The organization of the TES thus recapitulates the anatomical stratification of C4.4A expression we find in, for example, the esophageal epithelium [21]. As illustrated in Figures 3(g)–3(i), a multilayered* stratum granulosum* is also formed from day 6 to day 8 after exposure to the A/L interface, and this is accompanied by expression of Haldisin—the other epithelial differentiation biomarker belonging to the LU protein domain family. Keratinocytes from three different donors (4-day-old, 55- and 61-year-old) were used to produce TESs without affecting the C4.4A and Haldisin expression and localization.

### 3.3. C4.4A Expression in a Human Tissue-Engineered Wound Healing Model

**Figure 4:**
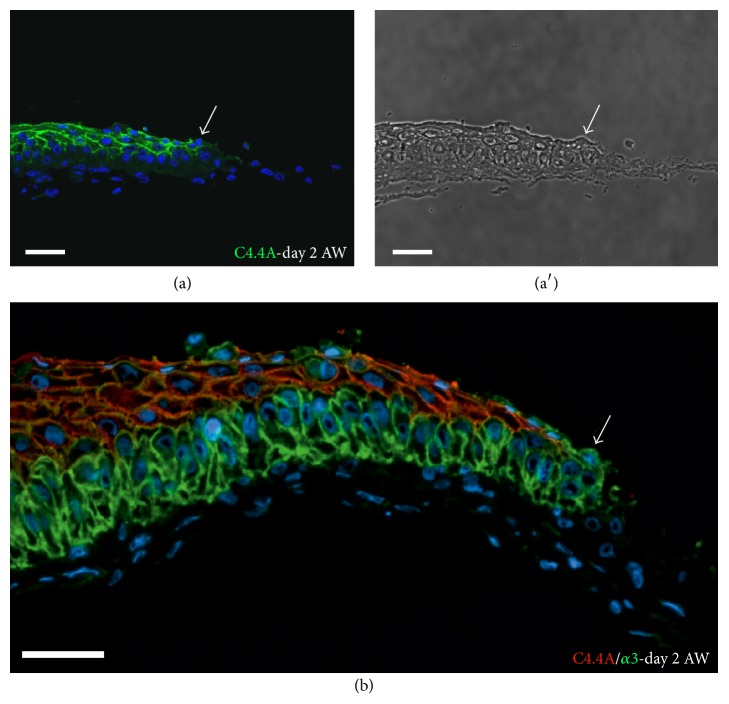
C4.4A in tissue-engineered wound healing model. (a-b) Tissue section of a human TES stained for C4.4A (green in (a) and red in (b)) and the *α*3 subunit of integrin (green in (b)) two days after the creation of a wound. Cell nuclei are stained with Hoechst (blue). Note that the foremost suprabasal keratinocytes of the migrating front that highly express the *α*3 subunit of integrin are devoid of C4.4A (arrows). (a′) Phase contrast image corresponding to panel (a). *α*3, *α*3 subunit of integrin; AW, after wound; TES, tissue-engineered skin. Scale bar = 50 *μ*m.

We next evaluated the expression of C4.4A in a wound healing model produced with human TES [33]. The distribution of the integrin *α*3*β*1, which is highly expressed in basal keratinocytes and which undergoes change in relative intensity at the tip of the migrating epithelial tongue during wound healing [38], was also evaluated. Upon wounding by a punch biopsy, keratinocytes at the margin of the wound start migrating on the extra fibroblast sheet upon which the wounded TES is placed, to close the wound. As illustrated in Figures 4(a) and 4(b), C4.4A was expressed suprabasally in the migrating epithelial tongue, and the labeling was negative in the basal keratinocytes that highly expressed the *α*3 subunit of integrin (Figure 4(b)). Interestingly, some foremost suprabasal keratinocytes at the tip of the migrating epithelial tongue were devoid of C4.4A (Figures 4(a) and 4(b), arrows), in agreement with our previous study of murine incisional skin wounds [15]. These C4.4A-negative suprabasal cells expressed the *α*3 subunit of integrin at the same level as basal cells (Figure 4(b), arrow).

## 4. Discussion

Given the expression pattern of the multidomain LU protein C4.4A in squamous epithelium, we have taken advantage of a surrogate model of human skin to investigate the expression kinetics of this protein in the genesis of a fully stratified epidermis. In line with previous observations in human skin [15], our results show that C4.4A is expressed in the spinous cells, of which one layer is already present at day 1 of culture at the A/L interface (Figure 2(a)). Over the next days of incubation, epidermal cells feed the development of a highly organized, stratified epithelium comprising several layers. At day 7, Haldisin expression was predominant in the upper cells of the granular layer of TESs in accordance with its expression pattern in mouse and human skin [13]. From day 1 to day 4, C4.4A expression exhibits a gradually more distinct membrane localization. This progression is paralleled in the corresponding in vivo process as studied in mouse embryogenesis, where C4.4A appears diffusely at embryonic day 14.5 (E14.5), when the spinous layer of the fetal back skin is about to develop in the approximately three-layered epithelium [39]. One day later, at E15.5, C4.4A presents with a strong membrane association [12]. Finally, the results obtained with the human tissue-engineered wound healing model, where the foremost migrating suprabasal keratinocyte is negative for C4.4A (Figure 4), comply with the in vivo situation as seen in murine incisional wounding [15].

It is well established that the basal-to-suprabasal switch taking place upon commitment to terminal differentiation of the epidermis is accompanied by a change in keratin expression from K5/14 in the basal cells to K1/10 suprabasally [40, 41]. As evident from the presented double immunofluorescence stainings, and inherently linked to the absence of C4.4A in the basal layer, the onset of C4.4A expression coincides with that of K10, indicating that C4.4A likewise could be a marker for this basal-to-spinous switch. In murine embryogenesis, C4.4A expression is also induced at the same time as K1/10 in the squamous epithelium of the nasal cavity and the vibrissal follicles and even a little earlier in the back and paw skin [12, 42, 43]. Furthermore, C4.4A has been reported to be transcriptionally regulated by the CCAAT/enhancer binding protein *β* [44], which can be induced by Notch, one of the main signals governing the basal-to-suprabasal switch [40].

In accordance with earlier studies, our observations support a role of C4.4A in early squamous differentiation. First, C4.4A is anatomically primarily localized to squamous epithelium, being absent in columnar epithelium [12, 21]. Second, treatment of murine skin with phorbol 12-myristate 13-acetate, which can commit cells to terminal cell division and ultimately squamous differentiation, entails an upregulation of C4.4A, restricted to the suprabasal, and not the basal, keratinocytes [15]. Third, the earliest appearance of C4.4A in embryogenic development of the esophagus occurs at E15.5 [12], corresponding to the initiation of esophageal transdifferentiation from a columnar to a stratified squamous epithelium [45]. Finally, C4.4A is found very early in the progression to malignant squamous cell carcinoma of the lung. While normal bronchial epithelium is devoid of C4.4A, it is, surprisingly, expressed in basal cell hyperplasia [19], which is a reactive change preceding the conversion into bronchial squamous metaplasia [46, 47], yet another process of transdifferentiation.

The functional consequences of this link to the squamous phenotype are, however, still unclear. Circumstantial evidence promotes the hypothesis of C4.4A being involved in cell-cell adhesion, which is rendered probable by the tethering of C4.4A to the cell membrane via a GPI-anchor [15]. The expression pattern of C4.4A is furthermore reminiscent to that of the well-characterized cell adhesion molecules E-cadherin [21] and *β*-catenin (Figure 1(c)). Our observations in monolayer culture of keratinocytes also fit this picture, with a clear membrane localization of C4.4A at cell-cell interaction points, suggesting a role of C4.4A in, for example, adherens junctions. In addition, the carbohydrate-binding lectin galectin-3, which has been implicated in cell-cell interactions and cell adhesion, has been identified as a ligand for C4.4A [48]. It is tempting to speculate that the abundant N- and O-linked glycosylation present in C4.4A [15] is targeted by the carbohydrate recognition domain of this lectin, with ensuing cross-linking of two neighboring cells [49].

It has already been clearly established that the self-assembly method of tissue engineering produces a skin substitute that to a very high degree mimics the anatomy of human skin, with the formation of the four characteristic epidermal cell layers that express well-established keratinocyte differentiation markers and a basement membrane at the dermal-epidermal junction [26]. The present results lend additional support to the applicability of this in vitro model for reporting on the corresponding in vivo situation, as illustrated by the tight regulation of the expression of C4.4A. A further strength of this substitute is that it is comprised of solely human components, which presents advantages as compared to the use of mouse models, given that it is not always biologically relevant to extrapolate results obtained in a murine system to the human counterpart. In the case of skin, there is an obvious species difference in the thickness of the epidermis, which might influence the mechanisms involved in epidermal development and wound healing.

## 5. Conclusions

In conclusion, we have demonstrated the potential utility of an in vitro surrogate model of human skin for performing functional studies on C4.4A and Haldisin, which ultimately could delineate the unknown biological role of these proteins. Such investigations might provide a framework for explaining our previous interesting finding of the high impact of C4.4A in the prognosis of patients with pulmonary adenocarcinoma [17, 20].

## Abbreviations

A/L: Air/liquid
DMEM: Dulbecco's modified Eagle's medium
E: Embryonic day
GPI: Glycosyl-phosphatidyl-inositol
K: Keratin
LU: Ly6/uPAR
OCT: Optimal cutting temperature
pAbs: Polyclonal antibodies
PBS: Phosphate buffer saline
TES: Tissue-engineered skin
uPAR: Urokinase-type plasminogen activator receptor.

## Additional Points

*Major Take-Home Points*. (i) C4.4A is located at cell-cell contact points between the more differentiated keratinocytes in monolayer culture. (ii) C4.4A expression emerges with the formation of a* stratum spinosum* layer in an in vitro developing human tissue-engineered skin model. (iii) After the creation of a wound, this tissue-engineered skin model recapitulates the dynamic expression of C4.4A as seen in the migrating suprabasal murine keratinocytes during wound healing. (iv) Tissue-engineered skin provides an optimal and convenient in vitro model for functional studies of C4.4A in human squamous epithelia.

## References

[1] Kjaergaard M., Hansen L. V., Jacobsen B., Gardsvoll H., Ploug M. (2008). Structure and ligand interactions of the urokinase receptor (uPAR). *Frontiers in Bioscience*.

[2] Loughner C. L., Bruford E. A., McAndrews M. S., Delp E. E., Swamynathan S., Swamynathan S. K. (2016). Organization, evolution and functions of the human and mouse Ly6/uPAR family genes. *Human Genomics*.

[3] Brodsky R. A. (2015). Complement in hemolytic anemia. *Blood*.

[4] Ploug M. (2013). Structure-driven design of radionuclide tracers for non-invasive imaging of uPAR and targeted radiotherapy. The tale of a synthetic peptide antagonist. *Theranostics*.

[5] Mysling S., Kristensen K. K., Larsson M. (2016). The angiopoietin-like protein ANGPTL4 catalyzes unfolding of the hydrolase domain in lipoprotein lipase and the endothelial membrane protein GPIHBP1 counteracts this unfolding. *eLife*.

[6] Mysling S., Kristensen K. K., Larsson M. (2016). The acidic domain of the endothelial membrane protein GPIHBP1 stabilizes lipoprotein lipase activity by preventing unfolding of its catalytic domain. *eLife*.

[7] Fujihara Y., Tokuhiro K., Muro Y. (2013). Expression of TEX101, regulated by ACE, is essential for the production of fertile mouse spermatozoa. *Proceedings of the National Academy of Sciences of the United States of America*.

[8] Wu Z., Liang R., Ohnesorg T. (2016). Heterogeneity of human neutrophil CD177 expression results from CD177P1 pseudogene conversion. *PLoS Genetics*.

[9] Adeyo O., Allan B. B., Barnes II R. H. (2014). Palmoplantar keratoderma along with neuromuscular and metabolic phenotypes in slurp1-deficient mice. *Journal of Investigative Dermatology*.

[10] Perez C., Khachemoune A. (2016). Mal de Meleda: a focused review. *American Journal of Clinical Dermatology*.

[11] Kriegbaum M. C., Persson M., Haldager L. (2011). Rational targeting of the urokinase receptor (uPAR): Development of antagonists and non-invasive imaging probes. *Current Drug Targets*.

[12] Kriegbaum M. C., Jacobsen B., Hald A., Ploug M. (2011). Expression of C4.4A, a structural uPAR homolog, reflects squamous epithelial differentiation in the adult mouse and during embryogenesis. *Journal of Histochemistry and Cytochemistry*.

[13] Gårdsvoll H., Kriegbaum M. C., Hertz E. P., Alpízar-Alpízar W., Ploug M. (2013). The urokinase receptor homolog Haldisin is a novel differentiation marker of stratum granulosum in squamous epithelia. *Journal of Histochemistry and Cytochemistry*.

[14] Jacobsen B., Ploug M. (2008). The urokinase receptor and its structural homologue C4.4A in human cancer: Expression, prognosis and pharmacological inhibition. *Current Medicinal Chemistry*.

[15] Hansen L. V., Gårdsvoll H., Nielsen B. S. (2004). Structural analysis and tissue localization of human C4.4A: A protein homologue of the urokinase receptor. *Biochemical Journal*.

[16] Rösel M., Claas C., Seiter S., Herlevsen M., Zöller M. (1998). Cloning and functional characterization of a new phosphatidyl-inositol anchored molecule of a metastasizing rat pancreatic tumor. *Oncogene*.

[17] Hansen L. V., Skov B. G., Ploug M., Pappot H. (2007). Tumour cell expression of C4.4A, a structural homologue of the urokinase receptor, correlates with poor prognosis in non-small cell lung cancer. *Lung Cancer*.

[18] Jacobsen B., Kriegbaum M. C., Santoni-Rugiu E., Ploug M. (2014). C4.4A as a biomarker in pulmonary adenocarcinoma and squamous cell carcinoma. *World Journal of Clinical Oncology*.

[19] Jacobsen B., Santoni-Rugiu E., Illemann M., Kriegbaum M. C., Lærum O. D., Ploug M. (2012). Expression of C4.4A in precursor lesions of pulmonary adenocarcinoma and squamous cell carcinoma. *International Journal of Cancer*.

[20] Jacobsen B., Muley T., Meister M. (2013). Ly6/upar-related protein c4.4a as a marker of solid growth pattern and poor prognosis in lung adenocarcinoma. *Journal of Thoracic Oncology*.

[21] Hansen L. V., Lærum O. D., Illemann M., Nielsen B. S., Ploug M. (2008). Altered expression of the urokinase receptor homologue, C4.4A, in invasive areas of human esophageal squamous cell carcinoma. *International Journal of Cancer*.

[22] Ohtsuka M., Yamamoto H., Masuzawa T. (2013). C4.4A expression is associated with a poor prognosis of esophageal squamous cell carcinoma. *Annals of Surgical Oncology*.

[23] Konishi K., Yamamoto H., Mimori K. (2010). Expression of C4.4A at the invasive front is a novel prognostic marker for disease recurrence of colorectal cancer. *Cancer Science*.

[24] Kriegbaum M. C., Clausen O. P. F., Lærum O. D., Ploug M. (2015). Expression of the Ly6/uPAR-domain proteins C4.4A and Haldisin in non-invasive and invasive skin lesions. *Journal of Histochemistry and Cytochemistry*.

[25] Kriegbaum M. C., Jacobsen B., Füchtbauer A. (2016). C4.4A gene ablation is compatible with normal epidermal development and causes modest overt phenotypes. *Scientific Reports*.

[26] Michel M., L'Heureux N., Pouliot R., Xu W., Auger F. A., Germain L. (1999). Characterization of a new tissue-engineered human skin equivalent with hair. *In Vitro Cellular and Developmental Biology—Animal*.

[27] Pouliot R., Larouche D., Auger F. A. (2002). Reconstructed human skin produced in vitro and grafted on athymic mice. *Transplantation*.

[28] Dubé J., Rochette-Drouin O., Lévesque P. (2010). Restoration of the transepithelial potential within tissue-engineered human skin in vitro and during the wound healing process in vivo. *Tissue Engineering Part A*.

[29] Robitaille H., Simard-Bisson C., Larouche D., Tanguay R. M., Blouin R., Germain L. (2010). The small heat-shock protein Hsp27 undergoes ERK-dependent phosphorylation and redistribution to the cytoskeleton in response to dual leucine zipper-bearing kinase expression. *Journal of Investigative Dermatology*.

[30] Germain L., Rouabhia M., Guignard R., Carrier L., Bouvard V., Auger F. A. (1993). Improvement of human keratinocyte isolation and culture using thermolysin. *Burns*.

[31] Bisson F., Rochefort É., Lavoie A. (2013). Irradiated human dermal fibroblasts are as efficient as mouse fibroblasts as a feeder layer to improve human epidermal cell culture lifespan. *International Journal of Molecular Sciences*.

[32] Larouche D., Paquet C., Fradette J., Carrier P., Auger F. A., Germain L. (2009). Regeneration of skin and cornea by tissue engineering. *Methods in Molecular Biology*.

[33] Laplante A. F., Germain L., Auger F. A., Moulin V. (2001). Mechanisms of wound reepithelialization: hints from a tissue-engineered reconstructed skin to long-standing questions. *The FASEB Journal*.

[34] Gårdsvoll H., Hansen L. V., Jørgensen T. J. D., Ploug M. (2007). A new tagging system for production of recombinant proteins in Drosophila S2 cells using the third domain of the urokinase receptor. *Protein Expression and Purification*.

[35] Royal I., Grenier A., Mailhot D., Marceau N. (1995). Polyomavirus middle T selective action on cytokeratin 14 gene expression in liver nonparenchymal epithelial cells. *Experimental Cell Research*.

[36] Larouche D., Cantin-Warren L., Desgagné M. (2016). Improved methods to produce tissue-engineered skin substitutes suitable for the permanent closure of full-thickness skin injuries. *BioResearch Open Access*.

[37] Moll R., Franke W. W., Schiller D. L., Geiger B., Krepler R. (1982). The catalog of human cytokeratins: patterns of expression in normal epithelia, tumors and cultured cells. *Cell*.

[38] Hertle M. D., Kubler M.-D., Leigh I. M., Watt F. M. (1992). Aberrant integrin expression during epidermal wound healing and in psoriatic epidermis. *Journal of Clinical Investigation*.

[39] Hanson J. (1947). The histogenesis of the epidermis in the rat and mouse. *Journal of Anatomy*.

[40] Blanpain C., Fuchs E. (2009). Epidermal homeostasis: a balancing act of stem cells in the skin. *Nature Reviews Molecular Cell Biology*.

[41] Fuchs E. (2009). Finding One's Niche in the Skin. *Cell Stem Cell*.

[42] Bickenbach J. R., Greer J. M., Bundman D. S., Rothnagel J. A., Roop D. R. (1995). Loricrin expression is coordinated with other epidermal proteins and the appearance of lipid lamellar granules in development. *Journal of Investigative Dermatology*.

[43] Byrne C., Tainsky M., Fuchs E. (1994). Programming gene expression in developing epidermis. *Development*.

[44] Fries F., Nazarenko I., Hess J., Claas A., Angel P., Zöller M. (2007). CEBP*β*, JunD and c-Jun contribute to the transcriptional activation of the metastasis-associated C4.4A gene. *International Journal of Cancer*.

[45] Yu W.-Y., Slack J. M. W., Tosh D. (2005). Conversion of columnar to stratified squamous epithelium in the developing mouse oesophagus. *Developmental Biology*.

[46] Leube R. E., Rustad T. J. (1992). Squamous cell metaplasia in the human lung: molecular characteristics of epithelial stratification. *Virchows Archiv B Cell Pathology Including Molecular Pathology*.

[47] Stosiek P., Kasper M., Moll R. (1992). Changes in cytokeratin expression accompany squamous metaplasia of the human respiratory epithelium. *Virchows Archiv A Pathological Anatomy and Histopathology*.

[48] Paret C., Bourouba M., Beer A. (2005). Ly6 family member C4.4A binds laminins 1 and 5, associates with Galectin-3 and supports cell migration. *International Journal of Cancer*.

[49] Nieminen J., Kuno A., Hirabayashi J., Sato S. (2007). Visualization of galectin-3 oligomerization on the surface of neutrophils and endothelial cells using fluorescence resonance energy transfer. *Journal of Biological Chemistry*.

